# Estimation of heterogeneous instantaneous reproduction numbers with application to characterize SARS-CoV-2 transmission in Massachusetts counties

**DOI:** 10.1371/journal.pcbi.1010434

**Published:** 2022-09-01

**Authors:** Zhenwei Zhou, Eric D. Kolaczyk, Robin N. Thompson, Laura F. White

**Affiliations:** 1 Department of Biostatistics, Boston University School of Public Health, Boston, Massachusetts, United States of America; 2 Department of Mathematics & Statistics, Boston University, Boston, Massachusetts, United States of America; 3 Department of Mathematics and Statistics, McGill University, Montreal, Canada; 4 Mathematics Institute and SBIDER, University of Warwick, Coventry, England, United Kingdom; University of Hong Kong, HONG KONG

## Abstract

The reproductive number is an important metric that has been widely used to quantify the infectiousness of communicable diseases. The time-varying instantaneous reproductive number is useful for monitoring the real-time dynamics of a disease to inform policy making for disease control. Local estimation of this metric, for instance at a county or city level, allows for more targeted interventions to curb transmission. However, simultaneous estimation of local reproductive numbers must account for potential sources of heterogeneity in these time-varying quantities—a key element of which is human mobility. We develop a statistical method that incorporates human mobility between multiple regions for estimating region-specific instantaneous reproductive numbers. The model also can account for exogenous cases imported from outside of the regions of interest. We propose two approaches to estimate the reproductive numbers, with mobility data used to adjust incidence in the first approach and to inform a formal priori distribution in the second (Bayesian) approach. Through a simulation study, we show that region-specific reproductive numbers can be well estimated if human mobility is reasonably well approximated by available data. We use this approach to estimate the instantaneous reproductive numbers of COVID-19 for 14 counties in Massachusetts using CDC case report data and the human mobility data collected by SafeGraph. We found that, accounting for mobility, our method produces estimates of reproductive numbers that are distinct across counties. In contrast, independent estimation of county-level reproductive numbers tends to produce similar values, as trends in county case-counts for the state are fairly concordant. These approaches can also be used to estimate any heterogeneity in transmission, for instance, age-dependent instantaneous reproductive number estimates. As people are more mobile and interact frequently in ways that permit transmission, it is important to account for this in the estimation of the reproductive number.

## Author Summary

To control the spreading of an infectious disease, it is very important to understand the real-time infectiousness of the pathogen that causes the disease. An existing metric called instantaneous reproductive number is often used to quantify the average number of secondary cases generated by individuals who are infectious at a certain time point, assuming no changes to current conditions. In practice, we might be interested in using the metric to describe the infectiousness in multiple regions. However, this is challenging when there are visitors traveling between these regions, since this could lead to a misclassification of where an individual is actually infected and create biased estimates for the instantaneous reproductive numbers. We developed a method that takes account of human mobility to estimate the instantaneous reproductive numbers for multiple regions simultaneously, which could reveal the heterogeneity of the metric. This method aims to provide helpful information on region-specific infectiousness for disease control measures that focus on the region with higher pathogen infectiousness. This approach is also applicable for estimating the reproductive number in the presence of other sources of heterogeneity, including by age.

This is a *PLOS Computational Biology* Methods paper.

## Introduction

In the aftermath of the pandemic caused by the SARS-CoV-1 virus, the idea of using surveillance data to estimate reproductive numbers was introduced and popularized by the seminal paper by Wallinga and Teunis [1]. Subsequent methods have been developed that are better suited to real-time estimation, particularly the approach to estimate the instantaneous reproductive number introduced by Fraser [2] and implemented in the popular EpiEstim R package [3, 4]. These methods have promised to be useful in surveillance and monitoring an epidemic, but as the pandemic caused by SARS-CoV-2 has demonstrated, there are still needed improvements to these approaches. Principal issues include accounting for reporting delays in the data, underreporting of cases, and heterogeneity in transmission by geography and by demographic factors, such as age. For these methods to be truly useful in the ongoing COVID-19 pandemic and for future events, these issues must be addressed. Work is being done on the first two issues. For instance, Li et al. [6], Gunther et al. [7] and Martinez et al. [8] propose solutions to the timeliness problem. Pitzer et al. [9] demonstrate the impact of reporting issues and White et al. [10] have shown how estimates of *R*(*t*) can be corrected with information on the reporting fraction of diseases. In this paper, we propose a framework for addressing heterogeneity in transmission, specifically due to human mobility, though our methods can be more generally applied.

Studies have shown that there is transmission heterogeneity in COVID-19, as well as other infectious diseases, which lead to a disproportional impact of the disease on some groups. Multiple studies have found strong evidence of strong heterogeneity wherein a small number of individuals are responsible for the vast majority of cases [11–13]. Additionally, in this COVID-19 pandemic, Sy et al. [14] have shown how mobility, such as subway usage, in NYC leads to disproportionate case burden among those who are not maintaining a physical distance. This implies it would be more efficient if we could account for the heterogeneity and focus control efforts on the populations with the highest transmission probabilities [15].

Many factors could contribute to the heterogeneity of virus transmission, including important systematic factors such as lower socioeconomic status (SES) that disadvantage certain groups and could lead to a higher probability of disease transmission. These factors often cluster geographically. The impact of these factors on virus transmission could be reflected in the reproductive numbers. Ideally, we will be able to discover the heterogeneity by examining the differences in the reproductive numbers between different regions. However, due to human mobility, the heterogeneity of the reproductive numbers among different regions could be masked. This is because human mobility could distribute the infectees by certain infectors to different regions, leading to misclassification of the incidence of one region to another.

We propose a framework for accounting for heterogeneity in disease transmission when estimating the time-varying instantaneous reproductive number for each region. This could help monitor changes in transmission to guide public health measures, for example, implementing more stringent disease control measures for the region with higher virus transmission. Our framework requires data to inform the source or patterns of heterogeneity. We focus on human mobility data to estimate the reproductive number for multiple regions or population groups. However, we note that the framework is suitable for understanding the effects of other important factors on the heterogeneity of the reproductive number, such as age.

We present an analytical framework with two approaches to estimate the dynamics of transmission heterogeneity. If we believe that the heterogeneity of the reproductive numbers can be recovered by accounting for population mixing due to human mobility, and we have confidence that the human mobility data represent the mixing of incidence, we suggest using an efficient and straightforward approach that adjusts the incidence prior to estimation. If instead we want to adjust for the importation of cases and more accurately quantify the uncertainty associated with the use of human mobility data with standard errors, we propose a more flexible and computationally intensive Bayesian approach that is more appropriate.

## Materials and methods

### Overview

We propose two approaches to estimate instantaneous reproductive numbers that incorporate human mobility data to account for heterogeneity. Both approaches are based on the framework of a system of renewal equations that bring human mobility into consideration. The difference between these two approaches is how the estimation handles potential uncertainty in the human mobility data. These methods can be applied to other types of heterogeneity, such as differential age-mixing where one might use the information on contact patterns between age groups. Our first approach simplifies the problem by assuming that human mobility data accurately represents the mixing patterns and corresponding incidence misclassification without error. In this setting, we propose an approach that extends the framework developed by Fraser et al. [2] to estimate the heterogeneous instantaneous reproductive numbers by adjusting the observed incidences for multiple regions using the human mobility data. In reality, there is likely some randomness in human mobility and we would typically wish to quantify the uncertainty due to other factors that might drive the heterogeneity of instantaneous reproductive numbers. For this setting, we use a system of renewal equations that incorporates human mobility data and estimate instantaneous reproductive numbers under a hierarchical Bayesian framework. Both approaches are evaluated by simulations, and implemented to estimate instantaneous reproductive numbers for all counties in Massachusetts, USA, during the COVID-19 pandemic together with human mobility data from SafeGraph.

### Data

The COVID-19 incidence data is provided by CDC case report [16] and we use incidence from July 2020 to March 2021 because testing and case reporting became more frequent and regular starting in July 2020. We aggregate confirmed cases in Massachusetts by date and county. Human mobility data is obtained from the multiscale dynamic human mobility flow dataset constructed and maintained by Kang et al. [17], who computed, aggregated and inferred the daily and weekly dynamic origin-to-destination (O-D) flow at three geographic scales (census tract, county and state) analyzing anonymous mobile phone users’ visits to various places provided by SafeGraph [18].

### Notation

Suppose that we want to estimate an instantaneous reproductive number, denoted as *R*(*t*), for *J* stratum, where the stratum can be geographical regions, age groups, communities, etc. Let *N*_*j*_(*t*), *t* = 1, …, *T* be the number of new cases reported at time *t* for region *j*, and *m*_*j*_(*t*) = *E*[*N*_*j*_(*t*)], where *t* = 1 is the first observation time and *T* is the last time with available data. The distribution of serial intervals is denoted as *ω*(*τ*|*θ*), where *τ* is the interval between the time of disease onset in an infector-infectee pair, and *θ* is the parameters of the distribution. There are several assumptions for both approaches that we propose:

1. Serial interval and reproductive number are statistically independent;
2. Reproductive number follows a Poisson distribution;
3. All infectors appear before those they infected;
4. Individuals mix homogeneously;
5. Closed population;
6. Complete case reporting;
7. Accurate mobility information;
8. The serial interval is the same as the reporting interval (i.e. the time between case report dates in an infector-infectee pair).

### Instantaneous reproductive number

The instantaneous reproductive number, originally developed by Fraser et al. [2], estimates the average number of secondary cases generated by individuals who are infectious at time *t* assuming no changes to current conditions. When using the instantaneous reproductive number, the expected incidence at time *t*, which is denoted as *m*(*t*), can be expressed as the following renewal equation:

$$\begin{array}{c} m\left(t\right)=\sum_{\tau <t}R\left(t\right)\omega \left(\tau \right)m\left(t-\tau \right). \end{array}$$ (1)

In practice, the estimator for *R*(*t*) can be computed with reported incidence *N*(*t*) as:

$$\begin{array}{c} \hat{R}\left(t\right)=\frac{N(t)}{\sum_{\tau <t}\omega \left(\tau \right)N\left(t-\tau \right)}. \end{array}$$ (2)

Cori et al. [3] used a Bayesian approach to estimate the *R*(*t*) with credible intervals and proposed smoothing the estimates by using a longer time window, assuming the *R*(*t*) stay the same within that window. Thompson et al. [4], and then Creswell et al. [5], developed extended versions of the method to perform estimation in the presence of imported cases. Based on the renewal Eq 1 as well as the estimation method developed by Cori et al. [3] and Thompson et al. [4], we can formulate the process into a system of renewal equations that incorporates the human mobility data, assuming that the mobility data accurately describe the how the infected individuals are travelling between regions.

Denote *P* as the *J*-by-*J* human mobility matrix that reclassifies incidences to the presumed location of the transmission event. Let *p*_*j*′ *j*_ be the entry of *P* matrix in the *j*′^*th*^ row and *j*^*th*^ column, and represents the proportion of population in region *j*′ that travels to region *j*. Then to describe incidences in multiple regions, we can extend the Eq (1) to a system of equations:

$$\begin{array}{c} m_{j}\left(t\right)=\sum_{j^{\prime }=1}^{J}[p_{j^{\prime }j}\left(t\right)\sum_{\tau <t}R_{j^{\prime }}\left(t\right)m_{j^{\prime }}\left(t-\tau \right)\omega \left(\tau \right)],\;\;j\in \left\{1,2,\ldots ,J\right\}, \end{array}$$ (3)

where $\sum_{j=1}^{j}p_{j^{\prime }j}\left(t\right)=1$.

Eq (3) describes the relationship between infectious individuals in region *j*′ at time *t* − *τ* and cases they infect who are reported in region *j* *τ* time points later. Two processes are at play, first the serial interval, *ω*(*τ*), as is commonly used in the EpiEstim estimator, which describes the probability of a secondary case taking *τ* time points to show up. Second, and unique to our formulation, is the *p*_*j*′ *j*_(*t*), which describes the probability of an individual infected at region *j*′ that travel to and be reported as a case in region *j* at time *t*. This formulation assumes that individuals have consistent mixing patterns between regions, e.g., regular commuting patterns, or that there is so-called slow-mixing, meaning that the individuals are traveling out of region *j*′ *τ* time points after they are infected.

We can rewrite Eq (3) in a matrix form, we have:

$$\begin{array}{c} m\left(t\right)=P^{⊤}\left(t\right)R\left(t\right)I_{J}\left(m\left(t-1\right),\ldots ,m\left(1\right)\right){\left(\omega \left(1\right),\ldots ,\omega \left(t-1\right)\right)}^{⊤}, \end{array}$$ (4)

where **m**(*t*) = (*m*_1_(*t*), *m*_2_(*t*), …, *m*_*J*_(*t*))^⊤^ is a vector of incidences for the *J* regions at time *t*, Therefore, (**m**(*t* − 1), …, **m**(1)) is a *J*-by-(*t* − 1) matrix for the incidences of *J* regions from time *t* − 1 to 1. **R**(*t*) = (*R*_1_(*t*), *R*_2_(*t*), …, *R*_*J*_(*t*))^⊤^ is a vector of instantaneous reproductive numbers for the *J* regions at time *t*. I_*J*_ is a *J*-by-*J* identity matrix.

Based on the above system of renewal equations, we propose two approaches for the estimation of heterogeneous *R*(*t*) incorporating mobility data as follows.

### Approach I—Incidence adjustment approach

In this approach, we use the matrix *P* from the human mobility data deterministically. According to Eq (4), assume that *P* is invertible, we have

$$\begin{array}{c} P^{-⊤}\left(t\right)m\left(t\right)=R\left(t\right)I_{J}\left(m\left(t-1\right),\ldots ,m\left(1\right)\right){\left(\omega \left(1\right),\ldots ,\omega \left(t-1\right)\right)}^{⊤}, \end{array}$$ (5)

Note that **m**(*t*) = (*m*_1_(*t*), *m*_2_(*t*), …, *m*_*J*_(*t*))^⊤^ = (*E*[*N*_1_(*t*)], *E*[*N*_2_(*t*)], …, *E*[*N*_*J*_(*t*)])^⊤^ = *E*[**N**(*t*)]. To estimate **R**(*t*) with the reported incidence **N**(*t*), let **N**_local_(*t*) = *P*^−⊤^(*t*)**N**(*t*), and assume $N_{j_{\text{local}}}\left(t\right)$ follows a Poisson distribution:

$$\begin{array}{c} \begin{array}{cc} & P(N_{j_{\text{local}}}\left(t\right)|N_{j}\left(t-1\right),\ldots ,N_{j}\left(1\right),\omega \left(t-1\right),\ldots ,\omega \left(1\right),R_{j}\left(t\right))\\ & =\frac{{\left(R_{j}\Lambda_{j}\left(t\right)\right)}^{N_{j_{\text{local}}}\left(t\right)}\text{exp}\left(-R_{j}\left(t\right)\Lambda_{j}\left(t\right)\right)}{N_{j_{\text{local}}}\left(t\right)!} \end{array} \end{array}$$ (6)

where Λ_*j*_(*t*) = ∑_*τ* < *t*_ *N*_*j*_(*t* − *τ*)*ω*(*τ*). $N_{j_{\text{local}}}\left(t\right)$ is a random variable for the incidence that actually should be attribute to region *j*, and the distribution of $N_{j_{\text{local}}}\left(t\right)$ is conditional on the previous reported cases in region *j*, the distribution of reporting interval *ω*(⋅) as well as the instantaneous reproductive number *R*_*j*_(*t*) in region *j* at time *t*. Λ_*j*_(*t*) is the cumulative reported cases in region *j* that contribute to the new reported cases at time *t*, Therefore, *R*_*j*_ Λ_*j*_(*t*) is the expectation of the random variable $N_{j_{\text{local}}}\left(t\right)$ that is assumed to follow a Poisson distribution.

Assume that *R*_*j*_(*t*) follows a gamma prior distribution *Gamma*(*a*, *b*), and within a *k*-days window (from day *t* − *k* + 1 to *t*), the incidences all depend on the same *R*_*j*_(*t*). we can write the posterior joint distribution of *R*_*j*_(*t*) as:

$$\begin{array}{cc} & P(R_{j}\left(t\right),N_{j_{\text{local}}}\left(t\right),\ldots ,N_{j_{\text{local}}}\left(t-k+1\right)|N_{j}\left(1\right),\ldots ,N_{j}\left(t-k\right)) \end{array}$$ (7a)

$$\begin{array}{cc} & \propto P\left(N_{j_{\text{local}}}\left(t\right)\ldots ,N_{j_{\text{local}}}\left(t-k+1\right)|R_{j}\left(t\right),N_{j}\left(1\right),\ldots ,N_{j}\left(t-k\right)\right)P\left(R_{j}\left(t\right)\right) \end{array}$$ (7b)

$$\begin{array}{cc} & =(\prod_{i=t-k+1}^{t}\frac{{\left(R_{j}\left(t\right)\Lambda_{j}\left(i\right)\right)}^{N_{j_{\text{local}}}\left(i\right)}}{N_{j_{\text{local}}}\left(i\right)!}\text{exp}\left(R_{j}\left(t\right)\Lambda_{j}\left(i\right)\right))\frac{R_{j}{\left(t\right)}^{a-1}}{\Gamma \left(a\right)b^{a}}\text{exp}(-\frac{R_{j}\left(t\right)}{b}) \end{array}$$ (7c)

$$\begin{array}{cc} & \propto R_{j}{\left(t\right)}^{a+\sum_{i}N_{j_{\text{local}}}\left(i\right)-1}\text{exp}(-R_{j}\left(t\right)(\sum_{i}\Lambda_{j}\left(i\right)+\frac{1}{b}))\prod_{i=t-k+1}^{t}\frac{\Lambda_{j}{\left(i\right)}^{N_{j_{\text{local}}}\left(i\right)}}{N_{j_{\text{local}}}\left(i\right)!} \end{array}$$ (7d)

Therefore, the posterior of *R*_*j*_(*t*) also follows a gamma distribution $Gamma{\left(a+\sum_{i}N_{j_{\text{local}}}\left(i\right)-1,\left(\sum_{i}\Lambda_{j}\left(i\right)+\frac{1}{b}\right)\right)}^{-1}$. The estimation can be performed by implementing the existing EpiEstim R package with the incidence adjustment data.

### Approach II—Bayesian approach

Based on the renewal equation with instantaneous reproductive number by previous studies [2, 19], we formulate the renewal equations for *J* regions as:

$$\begin{array}{c} m_{j}\left(t\right)=\mu_{j}\left(t\right)+\sum_{\tau <t}\sum_{j^{\prime }=1}^{J}R_{j^{\prime }j}\left(t\right)m_{j^{\prime }}\left(t-\tau \right)\omega \left(\tau \right),\;\;j\in \left\{1,2,\ldots ,J\right\}, \end{array}$$ (8)

where *μ*_*j*_(*t*) is the rate of exogenous infections (infections out of any of the regions *j* ∈ {1, …, *J*}) occurs in region *j*, and *ω*(*τ*) is the probability distribution of serial interval. We model *R*_*j*′ *j*_(*t*) = *R*_*j*′_(*t*)*p*_*j*′ *j*_(*t*), where *R*_*j*′_(*t*) is the region specific reproductive number for region *j*′ at time *t*, and *p*_*j*′ *j*_(*t*) represents the probability of an individual infected at region *j*′ that travel to and be reported as a case in region *j* at time *t*, assuming that {*p*_*j*′ *j*_(*t*):*j*′, *j* ∈ 1, 2, …, *J*} are known. Then we have:

$$\begin{array}{c} m_{j}\left(t\right)=\mu_{j}\left(t\right)+\sum_{j^{\prime }=1}^{J}[p_{j^{\prime }j}\left(t\right)\sum_{\tau <t}R_{j^{\prime }}\left(t\right)m_{j^{\prime }}\left(t-\tau \right)\omega \left(\tau \right)],\text{where}\;\;\sum_{j=1}^{j}p_{j^{\prime }j}\left(t\right)=1. \end{array}$$ (9)

*p*_*j*′ *j*_(t) here attempts to capture the mobility information of infected cases between the regions at time *t*, and we denote a matrix *P*(*t*) with entries *p*_*j*′ *j*_(*t*) as a transition matrix that models the infected subjects flowing across the regions. For example, while estimating *R*(*t*) for multiple regions, we can inform the *P*(*t*) matrix with mobility data between the regions and/or geographical distance between the regions. Within a Bayesian hierarchical modeling framework, Dirichlet priors for *P*(*t*) can incorporate prior knowledge for the estimation of *R*(*t*).

We model *R*_*j*′_(*t*) in (9) as:

$$\begin{array}{c} \text{log}\left(R_{j^{\prime }}\left(t\right)\right)=\beta_{j^{\prime }}\left(t\right)+\epsilon_{j^{\prime }},\;\;\epsilon_{j^{\prime }}∼N\left(0,\sigma_{j^{\prime }}\right), \end{array}$$ (10)

assuming *ϵ*_*j*′_ has constant variance over time.

Assume that the distribution of serial interval *ω*(*τ*) and *p*_*j*′ *j*_ is known; *N*_*j*_(*t*)∼*Poisson*(*m*_*j*_(*t*)) and {*N*_*j*_(*t*)} are independent conditional on *m*_*j*_(*t*), so we have the factorization: $P\left(\left\{N_{j}\left(t\right)\right\}|\left\{m_{j}\left(t\right)\right\}\right)=\prod_{j=1}^{J}P\left(N_{j}\left(t\right)|m_{j}\left(t\right)\right)$. Then we can sample the posterior distribution of parameters with Bayesian hierarchical modeling:

$$\begin{array}{cc} \text{log}(R_{j}\left(t\right)) & ∼N(\beta_{j}\left(t\right),\sigma_{j}), \end{array}$$ (11a)

$$\begin{array}{cc} m_{j}\left(t\right) & =\mu_{j}\left(t\right)+\sum_{j^{\prime }=1}^{J}[R_{j^{\prime }}\left(t\right)p_{j^{\prime }j}\left(t\right)\sum_{\tau <t}m_{j^{\prime }}\left(t-\tau \right)\omega \left(\tau \right)], \end{array}$$ (11b)

$$\begin{array}{cc} N_{j}\left(t\right) & ∼\text{Poisson}(m_{j}\left(t\right)),\;\;j=1,\ldots ,J, \end{array}$$ (11c)

with certain prior specifications for {*μ*_*j*_(*t*)}, {*β*_*j*_(*t*)}, {*σ*_*j*_}.

We also allow a smoothing window for the estimation of *R*_*j*_(*t*). If the length of the smoothing window is *k*, then we modify Eq (11c) to be:

$$\begin{array}{c} N_{j}\left(t^{\prime }\right)∼\text{Poisson}\left(m_{j}\left(t\right)\right),\;\;j=1,\ldots ,J,\;\;t^{\prime }=t,t+1,\ldots ,t+k-1. \end{array}$$ (12)

For Eq (12), we assume that the expected incidence at *k* consecutive time points are the same. Specifically, we assume that the incidence from time *t* to *t* + *k* − 1 follow the same Poisson distribution with mean *m*_*j*_(*t*).

### Assumption violation for mobility information

In practice, mobility information being used might not be accurate. In those scenarios, assuming that we have complete case reporting, we could have negative values in the local incidence according to the formula **N**_local_ = *P*^−⊤^(*t*)**N**(*t*). Since negative values would not make sense for the incidence as count data, it indicates that the mobility information that we are using is not accurate assuming case counts are accurate, which is an assumption violation for our method.

This is a violation of the assumption of accurate mobility information, indicating that it would be better to use higher quality mobility data that better describes how the infected individuals are traveling between regions. If we are not able to identify better mobility data, we propose an approach to adjust the P matrix that yields non-negative values for **N**_local_.

To adjust the *P* matrix, we use a shrinkage factor *s*_*j*_(*t*) For time *t* and region *j*. *s*_*j*_(*t*)∈[0, 1] describes the extent of shrinkage for the percentage of population flowing in or out of region *j* at time *t*. Denote the *P* matrix adjusted as *P*_adj_(*t*). To adjust *P* matrix so that the $N_{j_{\text{local}}}$ is non-negative, denote regions other than region *j* as {*j*′}, for the column of *P* matrix that correspond to region *j*, we let *P*_adj_(*t*)_(*j*′, *j*)_ = *s*_*j*_(*t*)*P*(*t*)_(*j*′, *j*)_ and *P*_adj_(*t*)_(*j*, *j*)_ = *P*(*t*)_(*j*, *j*)_ + (1 − *s*_*j*_(*t*))∑_*j*′ ∈ {1, …, *J*}/*j*_ *P*(*t*)_(*j*′, *j*)_, for the row of *P* matrix that correspond to region *j*, we let *P*_adj_(*t*)_(*j*, *j*′)_ = *s*_*j*_(*t*)*P*(*t*)_(*j*, *j*′)_ and *P*_adj_(*t*)_(*j*′, *j*′)_ = *P*(*t*)_(*j*′, *j*′)_ + (1 − *s*_*j*_(*t*))∑_*j*′ ∈ {1, …, *J*}/*j*_ *P*(*t*)_(*j*, *j*′)_. For each time point *t* and each region *j*, we search *s*_*j*_(*t*) from 1 to 0 with and interval of 0.01 until $N_{j_{\text{local}}}$ is non-negative. Since the regions with lower incidence are more readily impacted by the inaccurate mobility information, the adjustments are made such that a region with lower incidence will be adjusted first.

We further demonstrate how the proposed method to adjust *P* matrix could help in simulation scenario 4 where there is an inaccurate *P* matrix. We also emphasize that this hinges on the assumption that incidence data is accurate and that the approach we describe will not detect inaccuracies in the case where case counts are very large. This is important future work.

### Simulation

#### Simulation settings

**Scenario 1**: We consider three regions (*j* ∈ {*a*, *b*, *c*}), where there are no exogenous infections (except for the initial cases on day 0), so that *μ*_*j*_(*t*) = 0. Assuming *p*_*j*′ *j*_(*t*) and *ω*(*τ*) are known, we specify the 3-by-3 matrix *P*(*t*) = *P* with entries *p*_*j*′ *j*_(*t*) to be the same across time points, where *j*′ is row index and *j* is column index, and *P* is specified as the following:

$$P=(\begin{array}{ccc} 0.8 & 0.15 & 0.05\\ 0.2 & 0.6 & 0.2\\ 0.1 & 0.3 & 0.6 \end{array}),$$

and we generate discrete distribution *ω*(*τ*) for *τ* from the CDF of *f*(*τ*) = *Gamma*(2, 0.5):

$$\omega \left(\tau \right)=\{\begin{array}{cc} F(\tau )-F(\tau -1), & 0<\tau \leq 14\\ 0, & \tau >14 \end{array}$$

**Fig 1:**
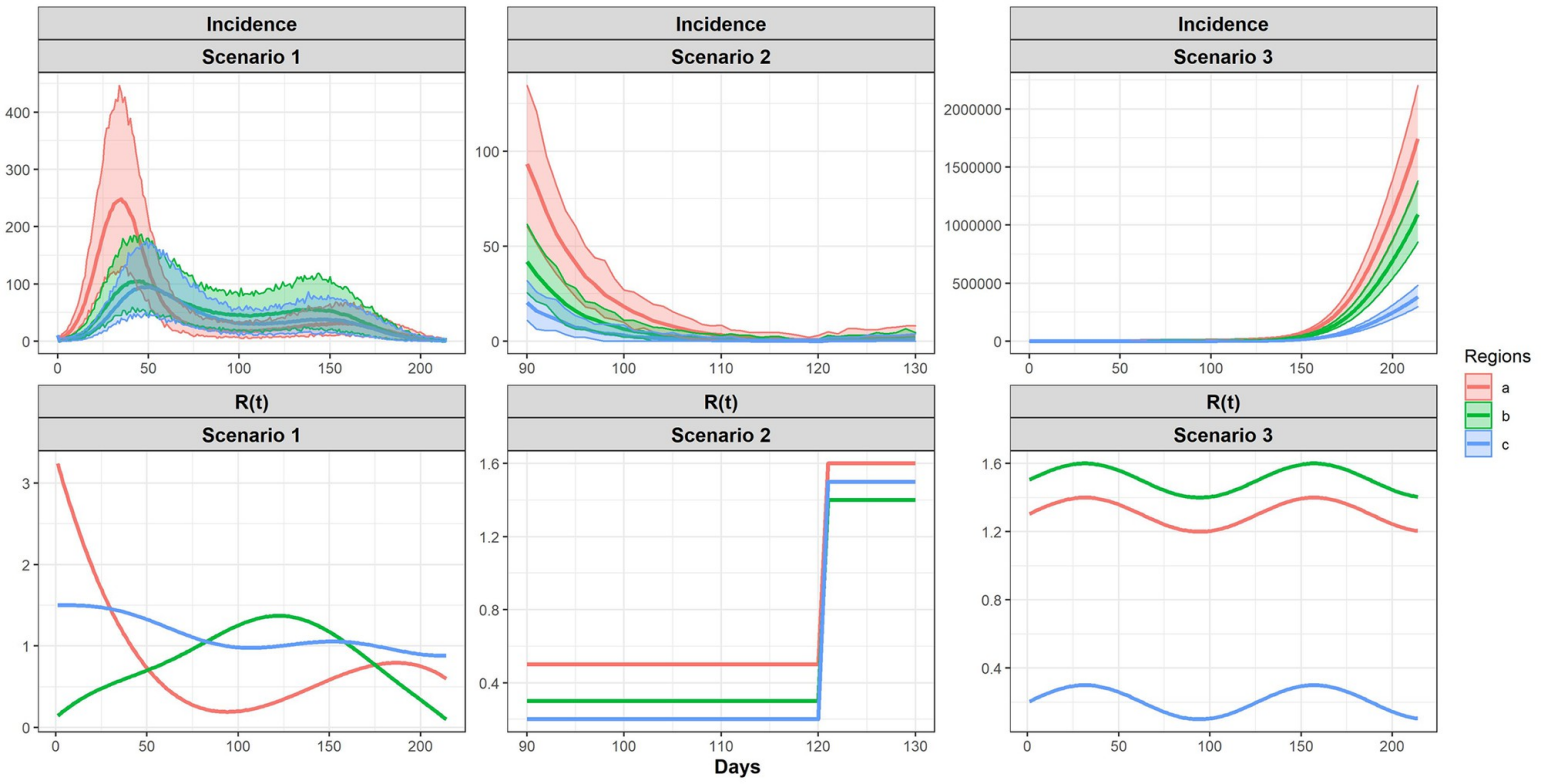
Specified *R*(*t*) functions and incidence for three regions from 100 replicates for simulation. The top panel shows the simulated incidence of 100 replicates. The shaded areas are the 95% quantile bands of the simulated incidences, the solid lines in the shaded area are the mean of the simulated incidences. The bottom panel shows the specified *R*(*t*) functions, which are plotted as solid lines.

For {*R*_*j*_(*t*)}, we specify nonlinear functions for each region (also shown in Fig 1):

$$\begin{array}{cc} R_{a}\left(t\right) & =(20cos\left(t/500\right)+{\left(\left(0.8t-50\right)\right)}^{2}-{\left(0.115t\right)}^{3})/1000+0.8\\ R_{b}\left(t\right) & =(30sin\left(t/150\right)+cos\left(t/20\right)-{\left(t/50\right)}^{2})/8-0.006t\\ R_{c}\left(t\right) & =(30cos\left(t/150\right)+2sin\left(t/20\right)+2{\left(t/50\right)}^{2})/20-0.005t \end{array}$$

To generate incidence data, we let the initializing cases to 10 in each region and let the maximum time of observation to be *T* = 214. According to Eq (9), with {*R*_*j*_(*t*)}, {*ω*(*τ*)} and matrix *P*(*t*), we can compute *m*_*j*_(*t*) and sample the incidences for *t* = 1, 2, …, *T* recursively. Note that we specified the same *P* for *P*(*t*) at all time points. Based on the incidences at previous time points *t*′ < *t*, and *R*_*j*_(*t*) at current time point *t*, we can compute *m*_*j*_(*t*) for the current time point *t*. Then the incidences for the current time point *t* are sampled from Poisson distribution with mean *m*_*j*_(*t*). The same steps are used to sample the incidence at the next time point *t* + 1, until we generated the incidences for *T* time points for each region. 100 Monte Carlo replicates are generated for the simulation study. Fig 1 shows the 100 replicates of simulated data.

We performed the incidence adjustment approach (Approach I) to estimate the instantaneous reproductive numbers on the simulated data described above. For the Bayesian approach (Approach II), we evaluated the performance of the model using different distribution assumption for the incidence *N*_*j*_(*t*), and also using different lengths of smoothing window. Then we explored whether using a prior for the transition *P* matrix to allow for more flexibility could yield proper estimates for *R*_*j*_(*t*). The performance of the proposed model is compared with the model without considering the heterogeneous of *R*_*j*_(*t*), that is using an identity *P* matrix.

For all models, we use *N*(0, 0.5) as the prior distribution for *β*_*j*_(*t*), and *N*(0, 1) for *σ*_*j*_, where *β*_*j*_(*t*) and *σ*_*j*_ are the hyperparameters for *R*_*j*_(*t*) in Eq (11a). Other model parameter settings are described below:

Model 1: constant *P* matrix, smoothing window is 1, assume Poisson distribution for *N*_*j*_(*t*);
Model 2: constant *P* matrix, smoothing window is 9, assume Poisson distribution for *N*_*j*_(*t*);
Model 3: constant *P* matrix, smoothing window is 9, assume Negative Binomial distribution with *ϕ* ∼ *N*(0, 5) for *N*_*j*_(*t*);
Model 4: random *P* matrix that each column follows a Dirichlet distribution centering at the true *P* matrix with large concentration parameter, smoothing window is 9, assume Poisson distribution for *N*_*j*_(*t*);
Model 5: constant identity *P* matrix, smoothing window is 9, assume Poisson distribution for *N*_*j*_(*t*) (this model is equivalent to Fraser’s method, which do not consider human mobility);

The estimates from Approach I (with and without mobility information) and model 4 and 5 of Approach II are shown in the main result section. Note that model 4 of Approach II is with mobility information, and model 5 of Approach II is without mobility information. Simulation results from Model 1, 2, 3 of Approach II are shown in S1 Appendix and S1 Fig.

**Scenario 2**: In practice, we might have a low count of cases for some of the regions, so we also evaluated the proposed approaches under the scenario where we have a lower count during a certain period of time. In the low count scenario, we specify the *R*(*t*) for the three regions to be three piece-wise functions, and it is shown in Fig 1.

$$\begin{array}{cc} R_{a}\left(t\right) & =1.2I(t\leq 80)+0.5I(80<t\leq 120)+1.6I(t>120)\\ R_{b}\left(t\right) & =1.4I(t\leq 80)+0.3I(80<t\leq 120)+1.4I(t>120)\\ R_{c}\left(t\right) & =I(t\leq 80)+0.2I(80<t\leq 120)+1.5I(t>120) \end{array}$$

We initiate with 50 incidences for the three regions, and simulated incidence from 100 replicates are shown in Fig 1. When performing estimation, we focus on the range from day 90 to day 130, because this range covers the period where the incidence decreases to zero or low counts that are close to zero. The result for estimated *R*(*t*)’s is shown in S1 Appendix and S2 Fig.

**Scenario 3**: We also evaluate the performance of the proposed approaches in a scenario where the population are traveling out of two of the regions with higher *R*(*t*) to the third region with lower *R*(*t*). We expect this scenario will show that if we do not consider the human mobility, we will overestimate the *R*(*t*) for the region where accepting population travel from other regions with higher *R*(*t*). In this scenario, we specify the *P* matrix to be:

$$P=(\begin{array}{ccc} 0.9 & 0 & 0\\ 0 & 0.9 & 0\\ 0.1 & 0.1 & 1 \end{array}).$$

The *P* matrix specified intends to create a scenario in which the population in region a and region b travel to region c, while the population in region c stay in place.

We specify the *R*(*t*) for the three regions to be (shown in Fig 1):

$$\begin{array}{cc} R_{a}\left(t\right) & =1.3+0.1sin(t/20)\\ R_{b}\left(t\right) & =0.5+0.1sin(t/20)\\ R_{c}\left(t\right) & =0.2+0.1sin(t/20) \end{array}$$

In this scenario, we initiate the 100 incidences for each of the three regions. We use Approach I to perform the estimation, since when the incidence counts are high, both approaches generate a similar result. The result of estimated *R*(*t*)’s is shown in S1 Appendix and S3 Fig. We also used another *P* matrix with higher population mixing between the regions, and the result of estimated *R*(*t*)’s is shown in S1 Appendix and S4 Fig.

**Scenario 4**: We show how the inaccurate *P* matrix might affect the estimates of **N**(*t*) and *R*(*t*) and evaluate the performance of the proposed method that adjust *P* matrix in this scenario. In this scenario, we specify the *P* matrix to be:

$$P=(\begin{array}{ccc} 0.995 & 0.2 & 0.2\\ 0.0025 & 0.6 & 0.2\\ 0.0025 & 0.2 & 0.6 \end{array}).$$

We use the *P* matrix above to generate data, then we apply approach I to the simulated data with an inaccurate *P* matrix with 10 times higher incidence exporting from region a to region b and c, the inaccurate *P* matrix is as below:

$$P_{\text{inaccurate}}=(\begin{array}{ccc} 0.95 & 0.2 & 0.2\\ 0.025 & 0.6 & 0.2\\ 0.025 & 0.2 & 0.6 \end{array}).$$

In this scenario, we initiate the 500 incidences for region a and 0 incidence for region b and c. With simulated incidences and the inaccurate *P* matrix, $N_{\text{local}}=P_{\text{inaccurate}}^{⊤}N$ yields negative values. We apply approach I with adjusted *P* matrix to demonstrate the performance of the proposed method to adjust the inaccurate *P* matrix for better estimates of *E*[**N**(*t*)]. We show the result of estimates for *E*[**N**(*t*)] and *R*(*t*) using true *P* matrix, inaccurate *P* matrix, adjusted inaccurate *P* matrix and identify *P* matrix (equivalent with not using mobility data) in S1 Appendix and S5 Fig.

### Real data application

We implement the two approaches described above to the COVID-19 incidence data from the CDC. Since case reporting was more regular starting around July 2020, we focus on the case report data from July 2020 to March 2021. We aim to estimate the heterogeneous instantaneous reproductive numbers for all counties (14 in total) in Massachusetts, USA.

Human mobility patterns across the counties are examined, followed by the estimation of instantaneous reproductive numbers as well as the expected incidence for each county. While performing the estimation, we assume the serial interval follows a gamma distribution Gamma(3.45, 0.66), which corresponds to a mean of 5.2 days and an SD of 2.8 days [20].

## Results

Our approaches are based on the renewal equation framework proposed by Fraser et al. [2] for estimating the instantaneous reproductive number. We extend the framework to incorporate human mobility data in a system of renewal equations to estimate the instantaneous reproductive numbers for multiple regions. We propose two approaches to carry out the estimation. For Approach I, we adjust the incidence in multiple regions according to the human mobility data and then estimate the instantaneous reproductive number separately in each region using the EpiEstim method. We call this the incidence adjustment approach. For Approach II, we perform estimation using a system of renewal equations in a hierarchical Bayesian framework. We call this the Bayesian approach.

In this section, we show results for both the simple incidence adjustment approach and the more complex system of renewal equations using simulation and data from Massachusetts during the COVID-19 pandemic.

### Simulation results

**Fig 2:**
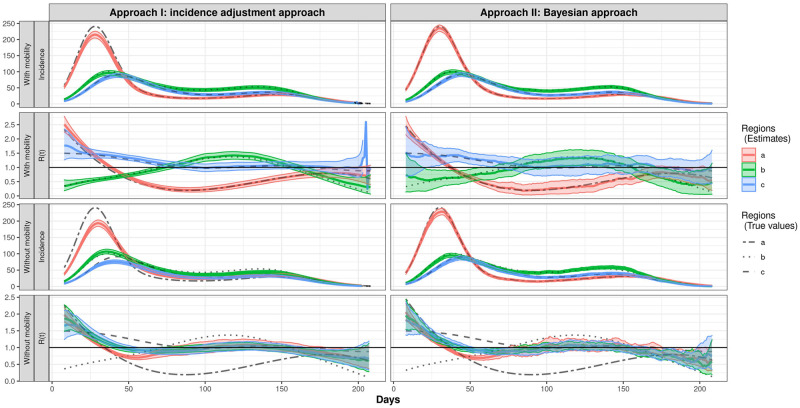
Estimated *E*[N(*t*)] and *R*(*t*) by region for Scenario 1. Solid lines are posterior means, along with the 95% credible bands (shaded). As noted at the sidebars on the left, the figures in the upper panel are the estimated Incidence and *R*(*t*) by region while using mobility data, and the lower panel shows the results from models without using mobility data. Both the results from Approach I and Approach II are provided.

Our simulation study considers three regions with substantially different transmission profiles over time, but reasonably similar patterns in incidence. The incidence data are simulated with pre-specified reproductive numbers over time as well as a transition matrix, which can be informed by mobility data in practice, that describes how the population in each region distribute to other regions. The details of the simulations are described in the Simulation Settings Section. Approach I is straightforward as we use the human mobility data deterministically to adjust the incidence. For Approach II, we evaluated the model using different assumptions on the distribution of incidence and randomness for the mobility data. In this section, we show the results from the two proposed approaches along with the naive approach that does not use mobility data in Fig 2. The naive approach estimates the reproductive numbers separately for each region, which is equivalent to Approach I and Approach II without using the mobility data. Other simulation results are in S1 Appendix.

Fig 2 shows the main result of the simulation. Table 1 summarizes the mean squared error (MSE), sensitivity and specificity for the estimates from Approach I and II with or without using mobility information. For Approach I, the incidence adjustment approach, a fixed transition matrix *P* (for mobility between regions) is used for the estimation. For Approach II, the Bayesian approach, Dirichlet priors with concentration parameter 10^4^ are placed on the row vectors in the transition matrix *P*. From Fig 2, we observe that both of Approach I and approach II provide estimated incidence for the 3 regions that are close to the incidence mean for 100 Monte Carlo replicates. The estimated reproductive numbers are also close to the true reproductive numbers. The credible bands of the estimated reproductive numbers are quite narrow for the incidence adjustment approach, while it is wider in the Bayesian approach. From Table 1, we see that the estimate of expectation of incidence and *R*(*t*) from Approach II is more accurate that Approach I in terms of MSE.

**Table 1:** Comparison of the Estimates of Expected Incidence and *R*(*t*) between Different Approaches under Scenario 1. Mean squared error (MSE) is computed as the squared error that averaged across days. Sensitivity and specificity are computed for the event *R*(*t*)>1. Note that Approach I without using mobility data is equivalent to the original Fraser’s method implemented in EpiEstim.

Approach	Estimate	Mobility	MSE	Sensitivity	Specificity
I	Incidence	With mobility	27.129	—	—
		Without mobility	137.490	—	—
	*R*(*t*)	With mobility	0.011	0.992	0.869
		Without mobility	0.146	0.543	0.723
II	Incidence	With mobility	3.091	—	—
		Without mobility	13.880	—	—
	*R*(*t*)	With mobility	0.005	0.960	0.912
		Without mobility	0.154	0.554	0.712

When we do not use mobility data (i.e. *P* is an identity matrix), the incidence estimates obtained by Approach I deviate from the true incidence mean, especially earlier in the outbreak, compared to that obtained by Approach II. Although the estimated incidences obtained by Approach II are close to the mean of simulated data, the *R*(*t*) estimates obtained by both approaches when not accounting for mobility, are very similar for each of the three regions and quite different from the true *R*(*t*) curves. The results show that the estimates for *R*(*t*) are close to the true *R*(*t*) if we use the mobility information in the model. But if we just stratify the data by region and estimate *R*(*t*) ignoring mobility patterns between regions, we are not able to capture the transmission differences. From Table 1, for estimated expectation of incidence from Approach I, the MSE is 27.129 when using mobility information and it is 137.49 when not using mobility information. For estimated *R*(*t*) from Approach I, the MSE is 0.011 when using mobility information, and it is 0.146 when not using mobility information. Both sensitivity and specificity that test for the event *R*(*t*)>1 are higher when using mobility information compare to not using mobility information. The pattern for the results from Approach II is similar to that from Approach I.

### COVID-19 results

#### Overview of incidence in Massachusetts

**Fig 3:**
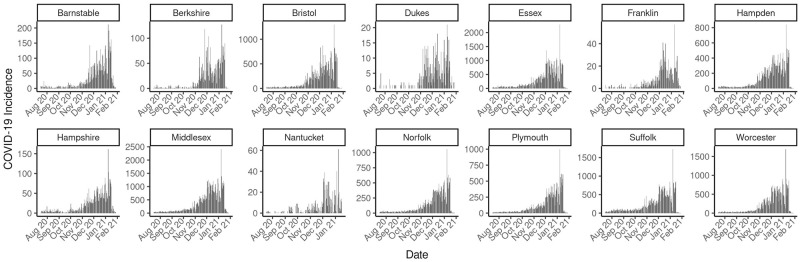
Reported Incidence for all MA counties. Incidence of COVID-19 from July 1^st^, 2020 to February 28^th^, 2021 for the 14 counties in the State of Massachusetts, USA.

Fig 3 is an overview of the incidence of COVID-19 from July 1^st^, 2020 to February 28^th^, 2021 for the 14 counties in the State of Massachusetts, USA. Essex, Middlesex and Suffolk county, the most populous counties, have relatively high incidence. The overall pattern of incidence across counties is similar, exhibiting an obvious increase after November 2020 during the second wave.

#### Population flow across counties in Massachusetts based on human mobility data

Human mobility data is obtained from the multiscale dynamic human mobility flow dataset constructed and maintained by Kang et al. [17]. They computed, aggregated and inferred the daily and weekly dynamic origin-to-destination (O-D) flow at three geographic scales (census tract, county and state) analyzing anonymous mobile phone users’ visits to various places provided by SafeGraph [18]. We use county-level data in Massachusetts for the modeling in this real data analysis. The human mobility data consists of the estimated number of visitors traveling from one county to another each day. For each county, we use the directional mobility data to compute the proportions of the population that travel to other counties as well as the proportion of the population that stays in the county. Therefore, we obtain the mobility matrices *P*(*t*) for each day. There are two assumptions we made in the analysis. First, we assume that the population is mixing slowly, or the population has a regular travel pattern, as described in Methods and Materials Section. Second, we assume that the mobility of the whole population is representative of the mobility of infected individuals. Both assumptions are important for the model as well as the analysis. If there is a large change in the travel pattern in the population, the first assumption will be violated, and the model will suffer due to the difficulty of tracing the location of the infected individuals over time. The second assumption makes it reasonable to infer the mobility of the infected individuals from the mobility of the population. The infected individuals, especially in the early stage of infection, could be more mobile than the general population, thus we consider our assumption to be conservative for the mobility of the infected individuals.

Before we analyze the real data with the proposed model, we first visualize the mobility data to examine the overall travel pattern of the population across counties in Massachusetts. We use *L*_*ij*_(*t*) to denote the number of visitors from county *j* to county *i* in day *t*. To visualize how the counties are clustered according to visitors traveling between them, we compute the average daily population flow $\frac{1}{T}\sum_{t=1}^{T}L_{ij}\left(t\right)$ for each (*i*, *j*) pair from July 1^st^, 2020 to February 28^th^, and stratify the flow by weekdays and weekends, assuming there will be different patterns for working days and non-working days.

**Fig 4:**
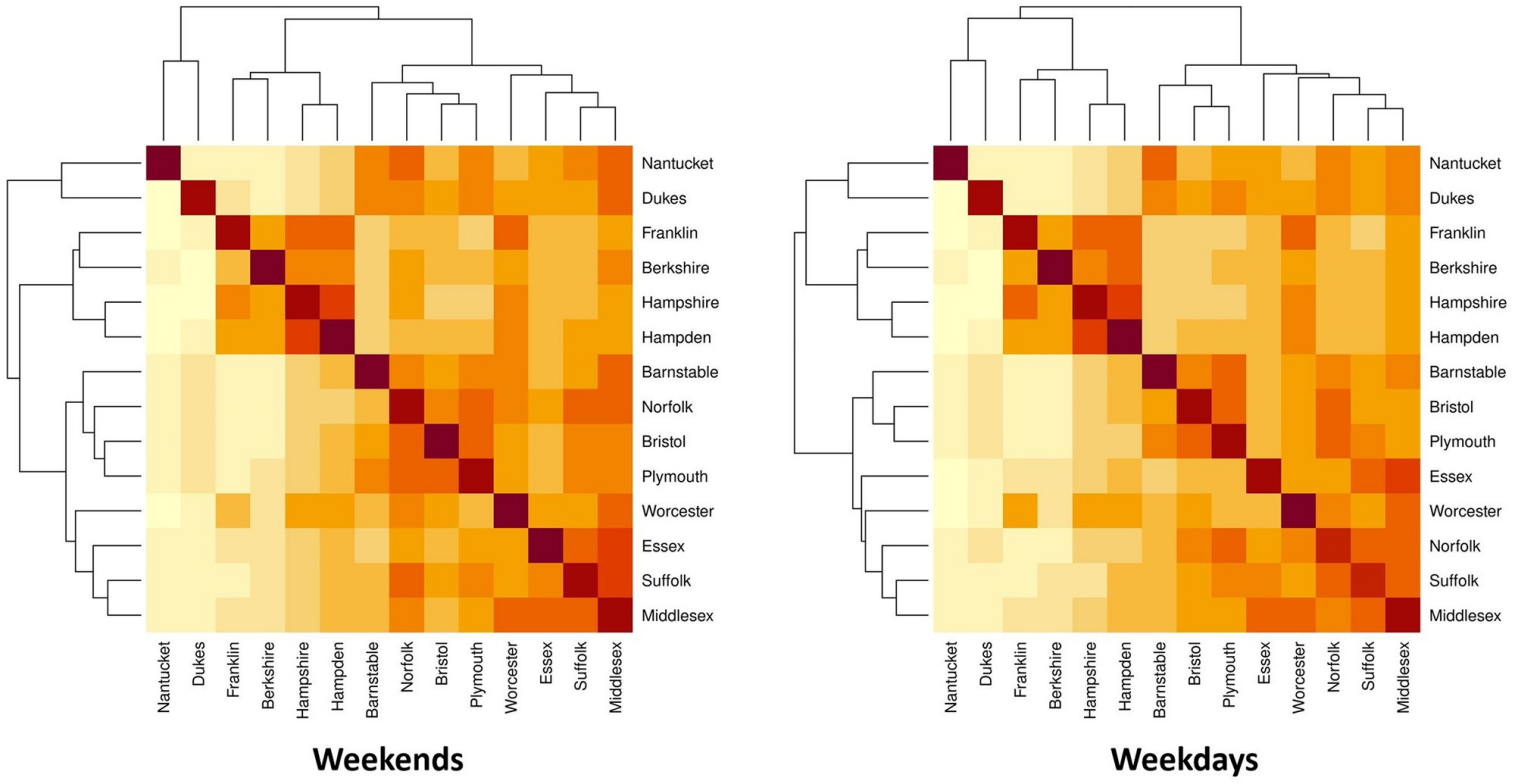
Heat maps for average population flow (log scaled) across regions during weekdays and weekends. Darker colors indicate regions with more flow between them.

**Fig 5:**
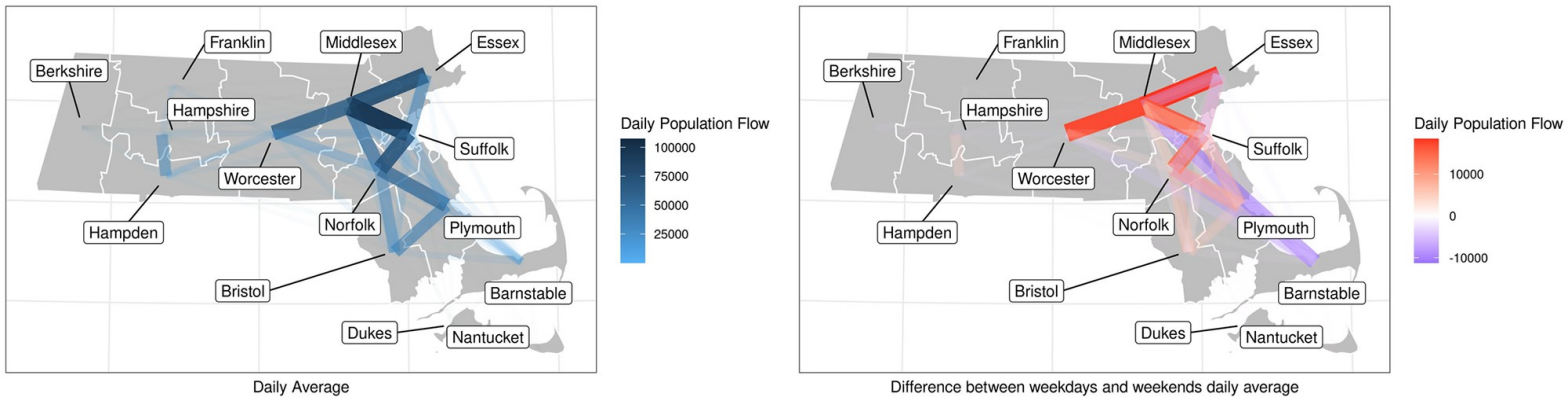
Human mobility network among counties of Massachusetts. The figure on the left shows the average daily population flow, and the figure on the right shows the difference of average population flow between weekdays and weekends, a positive value means the population flow is larger for weekdays than weekends, and a negative value means the population flow is smaller for weekdays than weekends. The maps are created in R and with urbnmapr and ggplot2 packages using data from the US Census Bureau, available at https://github.com/UrbanInstitute/urbnmapr.

There are notable differences between weekday and weekend patterns of mobility that can be seen in the heatmaps and dendrograms (Fig 4) generated with complete-linkage hierarchical clustering. During weekdays, most travel is between regions that are geographically proximate, for example, Barnstable, Bristol and Plymouth. On weekends, counties further apart are in the same cluster on the heat map, for example, Norfolk is in the cluster with Barnstable, Bristol and Plymouth. We also show the population flow on the geographical map in Fig 5. The clustering is more clear for regions that are geographically proximate for the daily average that is not stratified by weekdays and weekends. From the figure showing the difference between the weekdays’ daily average and weekends’ daily average, we observe that there is more of the population traveling between Essex, Worcester, Norfolk, Suffolk and Middlesex on weekdays compared to weekends, and less of the population traveling between Middlesex, Barnstable and Plymouth as well as between Norfolk, Barnstable and Plymouth. These patterns support the clustering in the heat maps.

Besides quantifying and visualizing the population flow between counties, quantifying the daily change of population for a county will help us understand the potential output of cases from that county and the infection pressure from other counties. We first compute population flow out and flow in before we compute population change. Assume that there are *J* counties considered, and the set J=(1,2,…,J) contains the indices for the *J* counties. For county j∈J, to compute the population flow out in day *t*, we can aggregate the number of visitors from county *j* that travel to other counties to be the size of population flow out for county *j* in day *t*, and denote it as $L_{\text{out}}^{\left(j\right)}\left(t\right)=\sum_{i\in J/j}L_{ij}\left(t\right)$. Also, we can aggregate the number of visitors from other counties that travel to county *j* to be the size of population flow in county *j* in day *t*, and denote this as $L_{\text{in}}^{\left(j\right)}\left(t\right)=\sum_{k\in J/j}L_{jk}\left(t\right)$. And we use *L*(*t*)^(*j*)^ to denote the population size of county *j* on day *t*. Note that the data is for human mobility in that day, instead of a permanent move.

**Fig 6:**
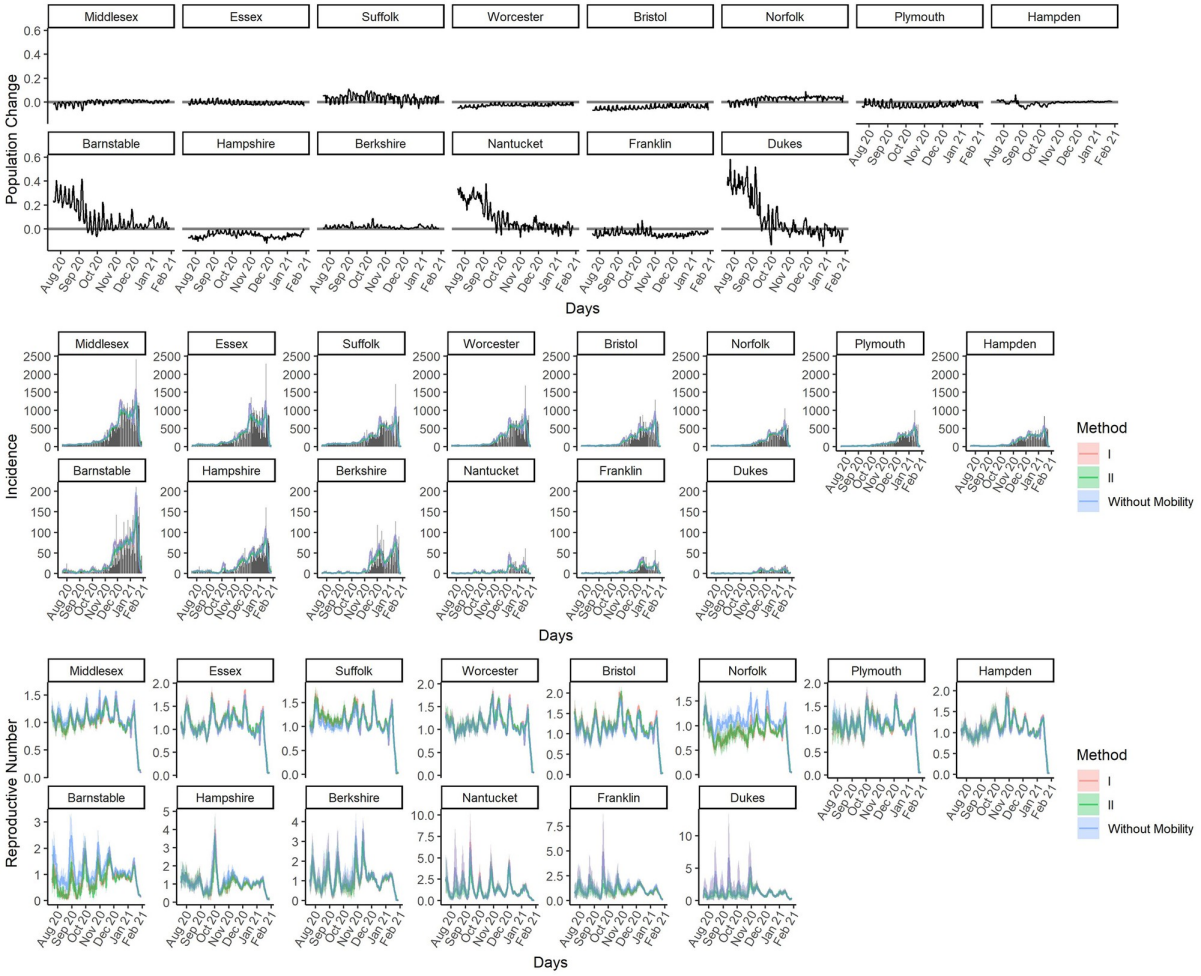
Population change, estimated Incidence and *R*(*t*) for all MA counties. Solid lines are the posterior means for incidence and *R*(*t*), along with the 95% credible band. The bar plots for the observed incidence are also shown. Results from Approach I, the incidence adjustment approach, are in red and those from Approach II, the Bayesian approach, are in green, and those from the original Fraser’s method (obtained by Approach II without incorporating mobility data) are in blue.

Population change, which is denoted as $L_{\text{change}}^{\left(j\right)}\left(t\right)$, can inform how the population in region *j* is mixing with other regions in day *t*. Population change refers to the change in population size in day *t* compared to the population size in day *t* − 1 as a ratio, that is $L_{\text{change}}^{\left(j\right)}\left(t\right)=\frac{L_{\text{in}}^{\left(j\right)}\left(t\right)-L_{\text{out}}^{\left(j\right)}\left(t\right)}{L^{\left(j\right)}\left(t-1\right)}$, where $L_{\text{in}}^{\left(j\right)}\left(t\right)$ denotes the size of population flow in and $L_{\text{out}}^{\left(j\right)}\left(t\right)$ the size of population flow out in day *t* for county *j*. We examine the percentage of population change for all counties shown as the top panel in Fig 6. The population change plot shows that there is a relatively high percentage of population change for Barnstable, Dukes and Nantucket before October, 2020, due to the population inflow.

#### Estimated expected incidence and heterogeneous instantaneous reproductive numbers

We applied both Approach I and II to the COVID-19 incidence data of 14 counties in the State of Massachusetts, USA, while taken the mobility data into account. The middle and bottom panel in Fig 6 shows the estimated expected incidence and *R*(*t*) for all counties with both of our proposed methods. Results from Approach I, the incidence adjustment approach, are shown in red and those from Approach II, the Bayesian approach, are shown in green, and the results from original Fraser’s approach (obtained by Approach II without incorporating mobility data) are shown in blue.

When using mobility information, the estimated *R*(*t*)’s are relatively lower for Barnstable and Norfolk compare to that when not using mobility information. Barnstable has a high percentage of population flow in from July to October, and during that time period, *R*(*t*) estimates are clearly lower to that when not using mobility information. For Norfolk, the inflow of population happens after August, and we observed lower estimates of *R*(*t*) when using mobility information compare to that when not using mobility information. From the result, it is possible that the increase of incidence in these counties is due to inflow of incidences from other counties with higher *R*(*t*). From the simulation with scenario 3, we demonstrated that if there is a region with lower *R*(*t*) and accepting incidences from other regions with higher *R*(*t*), we will overestimate the *R*(*t*) if we do not consider the mobility data.

The results from Approach II are similar to those from Approach I when incidences are high, while in counties with lower reproductive numbers the estimated *R*(*t*)’s from Approach II are smaller than that from Approach I. The difference of estimates between these two approaches could be due to the low count of incidence. In simulation scenario 2, we observe that Approach I tends to overestimate R(t) compared to Approach II in presence of a low incidence count.

## Discussion

It is well-established for many infectious diseases that there is substantial heterogeneity in transmission patterns. One might reasonably expect that some of this variability occurs geographically due to a potentially complex combination of social factors and some amount of stochastic effects. Estimating spatially granular reproductive numbers allows for greater targeting of interventions and the potential to uncover the factors that drive heightened transmission. We have described two approaches for estimating *R*(*t*) that incorporate mixing patterns between distinct groups, which in our setting is informed by mobility data between geographic regions.

We demonstrate how these two approaches perform on simulated data. Simulations shows that both of the approaches are able to estimate the heterogeneous instantaneous reproductive numbers for multiple regions well when the mobility data is well-specified. We observed that the second approach has larger variability. This is expected since in Approach II we incorporate some of our uncertainty around the accuracy of the mobility data, allowing some flexibility in the case where the mobility data might not exactly represent how incident cases are flowing between the regions. This means that the first approach is likely more sensitive to inaccuracies in the mobility data, while the second approach samples over for the mobility prior together with the other parameters, allowing for some misspecification. Therefore, if we have high-quality mobility data that is representative of the population and incidence flow, and are only interested in obtaining reproductive numbers for multiple regions, we can use the more efficient Approach I. If we want to incorporate uncertainty in the mobility data and/or investigate factors that are associated with *R*(*t*), we can use Approach II.

In our simulation, we show that using mobility information allows us to obtain estimates for *R*(*t*) that are close to the true *R*(*t*) and that this is not feasible when mobility data is not used (see scenario 1). In other words, simply stratifying data by region and estimating *R*(*t*) ignoring mobility patterns between regions does not appropriately capture transmission differences. This is especially important when there are regions with a lower *R*(*t*) accepting population flow from regions with a higher *R*(*t*). For example, people might live in counties with lower *R*(*t*), but work in counties with higher *R*(*t*). If mobility information is not taken into account, we could overestimate *R*(*t*) for the counties in which these people are living, and underestimate the *R*(*t*) in the counties where they work. This is shown in our simulation results (see scenario 3).

A potential additional benefit of the more computationally intensive second approach is that local factors, such as age, socioeconomic status and disease containment policies can be incorporated into the estimation framework. This can potentially allow one to not only estimate more accurately the differences between regions, but also potentially start to more carefully understand some of the underlying factors influencing the transmission differences.

When we consider the dynamics of COVID-19 in Massachusetts, the county-level results show that the two approaches yield similar estimates, but that these are distinct from the naive approach that ignores mobility. Generally, the estimated incidence data is similar, but there are some differences in the estimated *R*(*t*)’s with mobility incorporated. *R*(*t*) estimates from Approach I have a larger credible band for the counties with lower incidence, such as Nantucket, Franklin and Dukes. The second approach produces smoother estimated *R*(*t*)’s when incidences are low. From simulation scenario 2, we have shown that Approach I tends to overestimate *R*(*t*) compared to Approach II when there are low counts for incidence. This could be the reason for the larger *R*(*t*) estimates from Approach I for Nantucket, Franklin and Dukes during the time with low incidence count. For Barnstable and Norfolk, we observe a positive population change that correspond to lower estimated *R*(*t*) from Approach I and II when compare with not using mobility information, this might due to the incidence input from other counties with higher *R*(*t*).

For both of the methods, an important assumption is that the mobility data describes the flow of infectious individuals, even though it is not explicitly measuring this. This might not hold if individuals dramatically change their behavior when they are infectious. A potential approach to cope with this problem might be adding parameters informed by behavioral data among infectious individuals as weights to the mobility data to account for changed mobility due to the disease.

In a summary, the instantaneous *R*(*t*) is an important metric for infectious disease surveillance, since it provides a real-time description of the transmission dynamics among the population. While estimating *R*(*t*) for multiple regions, we expect to see heterogeneity. However, estimating the heterogeneity can be challenging when there is extensive population flow between regions leading to a mixing of the population that can mask or misrepresent the true transmission dynamics. We have presented two methods that incorporate mobility data for the estimation of spatially heterogeneous *R*(*t*). The ultimate goal of this approach is to identify the regions with the higher transmission in which to focus interventions as well as study potential mechanisms of transmission. These methods have broad applicability to estimating *R*(*t*) in the presence of any potential heterogeneities, such as age-mixing which can use mixing behavior described by contact surveys such as those performed by Mossong et al. [21].

## Supporting information

S1 Appendix Other simulation results. Simulation result for Model 1, 2 and 3 in scenario 1, and results for scenario 2, 3 and 4.
(PDF)

S1 Fig Estimated *E*[N(*t*)] and *R*(*t*) by Model 1, 2 and 3. Solid lines are posterior means, along with the 95% credible bands (shaded). The results are summarized from Approach II with different parameter settings described in the Simulation Settings Section.
(TIF)

S2 Fig Estimated *R*(*t*) for low incidence scenario. Solid lines are posterior means, along with the 95% credible bands (shaded). The results are summarized from Approach I and Approach II.
(TIF)

S3 Fig Estimated *R*(*t*) for population input from other regions. Solid lines are posterior means, along with the 95% credible bands (shaded). The results are summarized from Approach I with and without incorporating mobility information.
(TIF)

S4 Fig Estimated *R*(*t*) for population input from other regions with higher population mixing among the regions. Solid lines are posterior means, along with the 95% credible bands (shaded). The results are summarized from Approach I with and without incorporating mobility information.
(TIF)

S5 Fig Estimated *E*[N(*t*)] and *R*(*t*) in scenario with inaccurate *P* matrix. Solid lines are posterior means, along with the 95% credible bands (shaded), the color of solid lines represents the mobility information used in the model. Red dashed lines are means of **N**(*t*) and *R*(*t*) in the simulated data.
(JPG)
