# Supplementary material for: Estimation of heterogeneous instantaneous reproduction numbers with application to characterize SARS-CoV-2 transmission in Massachusetts counties
Source: PLoS Comput Biol. 2022 Sep 1;18(9):e1010434. doi: 10.1371/journal.pcbi.1010434 (PMC9473631; doi:10.1371/journal.pcbi.1010434)
Supplement: S1 Appendix — Simulation result for Model 1, 2 and 3 in scenario 1, and results for scenario 2, 3 and 4. (PDF) [file pcbi.1010434.s001.pdf]

Estimation of heterogeneous instantaneous reproduction numbers with application to characterize SARS-CoV-2 transmission in Massachusetts counties

Zhenwei Zhou[1**], Eric D. Kolaczyk[2,3], Robin N. Thompson[4], Laura F. White[1*]

1 Department of Biostatistics, Boston University School of Public Health, Boston, Massachusetts, US
2 Department of Mathematics & Statistics, Boston University, Boston, Massachusetts, US
3 Department of Mathematics and Statistics, McGill University, Montreal, Canada
4 Mathematics Institute and SBIDER, University of Warwick, Coventry, England, UK

* lfwhite@bu.edu
** zwzhou@bu.edu

Supporting information

Simulation result for Model 1, 2 and 3 in scenario 1

Fig S1 shows the estimated incidence and $R(t)$'s by model 1, 2 and 3. In model 1, the incidences are assumed to be following Poisson distribution. The estimated $R(t)$'s have the trend that align with the true $R(t)$ curves simulated. And the predicted incidence for the 3 regions are close with the mean of incidence in the simulated datasets. Since there is no smoothing for the estimates in model 1, we also observe more variations of the estimates within a short period of time.

In model 2, we assume the incidences are following negative binomial distributions although the simulated data is from Poisson distribution. The credible bands are wider than that in results from model 1 and the estimates are also not smooth as that in results from model 1.

In model 3, incidences are assumed to be following Poisson distribution. The trend of $R(t)$ estimates are also aligning with the true $R(t)$, and the estimates are smoother with a smoothing window of 8 days in the model. The credible bands of the posterior estimates are narrower.

Fig S1. Estimated $E[\mathbf{N}(t)]$ and $R(t)$ by Model 1, 2 and 3. Solid lines are posterior means, along with the 95% credible bands (shaded). The results are summarized from Approach II with different parameter settings described in the Simulation Settings Section.

Simulation result for scenario 2: low incidence count

Fig S2. Estimated $R(t)$ for low incidence scenario. Solid lines are posterior means, along with the 95% credible bands (shaded). The results are summarized from Approach I and Approach II.

Fig S2 shows the estimated $R(t)$'s for the three regions by both Approach I and Approach II. Both of the approaches have a wider credible band when the incidence counts are low from day 110 to day 130. For Approach I, the estimated $R(t)$ are much larger than the true $R(t)$ with low incidence counts, for example, for region a, the posterior mean of $R(t)$ is 5.8 and the true $R(t)$ is 1.6 at day 125. For Approach II, the estimated $R(t)$ is closer to the true $R(t)$, for example, for region a, the posterior mean of $R(t)$ is 1.72 and the true $R(t)$ is 1.6 at day 125.

Simulation result for scenario 3: population input from other regions

Fig S3. Estimated $R(t)$ for population input from other regions. Solid lines are posterior means, along with the 95% credible bands (shaded). The results are summarized from Approach I with and without incorporating mobility information.

In the scenario where the population travel from two regions with higher $R(t)$ to the third region with a lower $R(t)$, from Fig S3, we observed the difference for the estimated $R(t)$ when using Approach I with mobility information and without mobility information, note that Approach I without mobility information is equivalent to the original Fraser's method. There is an overestimate for the $R(t)$ in the region accepting population from the other two regions.

We used another P matrix with higher population mixing to see how this could impact the performance in this scenario. The P matrix we specified is:

$$P = \begin{pmatrix} 0.8 & 0 & 0.2 \\ 0 & 0.7 & 0.3 \\ 0.1 & 0.1 & 0.8 \end{pmatrix}.$$

Fig S4. Estimated $R(t)$ for population input from other regions with higher population mixing among the regions. Solid lines are posterior means, along with the 95% credible bands (shaded). The results are summarized from Approach I with and without incorporating mobility information.

Fig S4 shows the results from the simulation that we used a P matrix with higher population mixing. A pattern similar to that shown in Fig S3 is observed, we can see that the estimates of $R(t)$ from the model that uses mobility data are close to the true $R(t)$, but the estimates of $R(t)$ for region c is overestimated by the model that does not use the mobility data.

Simulation result for scenario 4: assumption violation for mobility information

Fig S5. Estimated $E[\mathbf{N}(t)]$ and $R(t)$ in scenario with inaccurate P matrix. Solid lines are posterior means, along with the 95% credible bands (shaded), the color of solid lines represents the mobility information used in the model. Red dashed lines are means of $\mathbf{N}(t)$ and $R(t)$ in the simulated data.

In scenario 4, there are high incidences in region a, and relatively low incidences in region b and c. As shown in Fig S5, when using the true P matrix, the estimates of $E[\mathbf{N}(t)]$ and $R(t)$ are close to the means of $\mathbf{N}(t)$ and $R(t)$ in the simulated data. When

we use the inaccurate P matrix with much higher exportation of incidences from region a to b and c, we observe overestimation for $E[\mathbf{N}(t)]$, and the estimates of $R(t)$ are not accurate. When the adjusted P matrix is used, the estimates of $E[\mathbf{N}(t)]$ become close to the means of $\mathbf{N}(t)$ and $R(t)$ in the simulated data. The estimates of $R(t)$ is better than that without adjusting the inaccurate P matrix. Although using the adjustment of P matrix could not compare with using a true P matrix, the pattern for $R(t)$ is aligning better than the estimated $R(t)$ without considering mobility information.
