# Supplementary figures and images for: Estimation of heterogeneous instantaneous reproduction numbers with application to characterize SARS-CoV-2 transmission in Massachusetts counties
Source: PLoS Comput Biol. 2022 Sep 1;18(9):e1010434. doi: 10.1371/journal.pcbi.1010434 (PMC9473631; doi:10.1371/journal.pcbi.1010434)

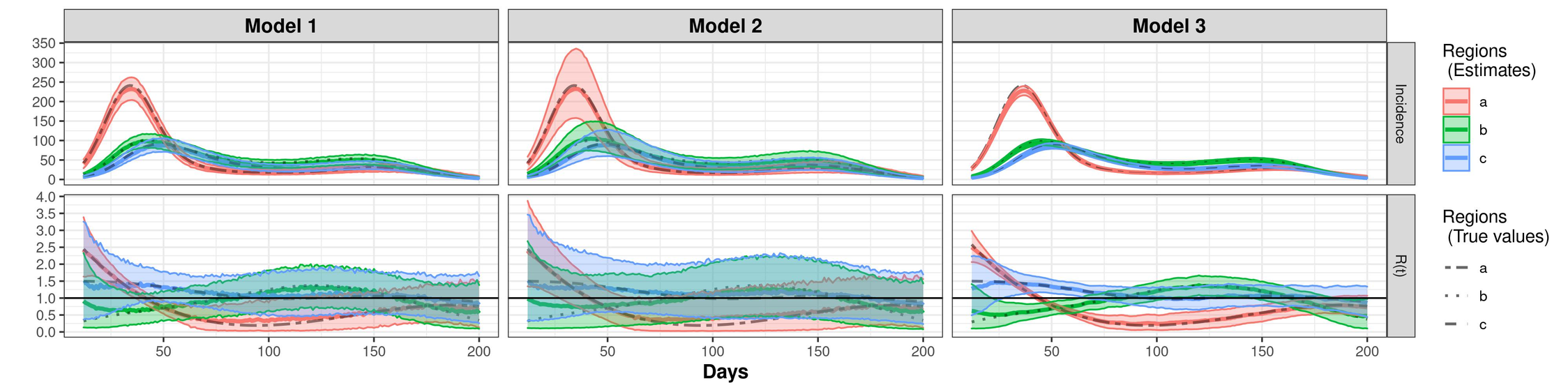

Supplement: S1 Fig — Solid lines are posterior means, along with the 95% credible bands (shaded). The results are summarized from Approach II with different parameter settings described in the Simulation Settings Section. (TIF) [file pcbi.1010434.s002.tif]

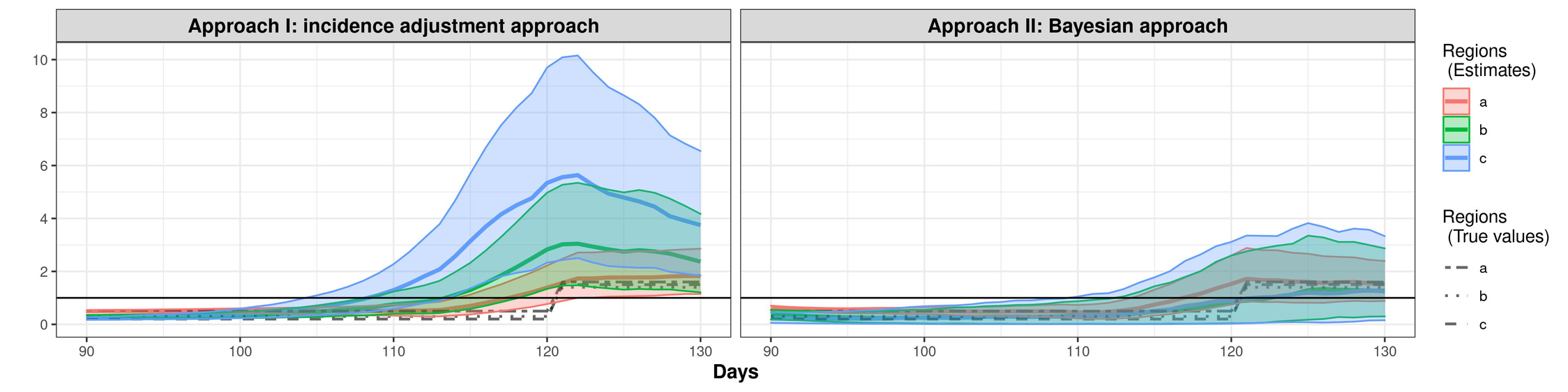

Supplement: S2 Fig — Solid lines are posterior means, along with the 95% credible bands (shaded). The results are summarized from Approach I and Approach II. (TIF) [file pcbi.1010434.s003.tif]

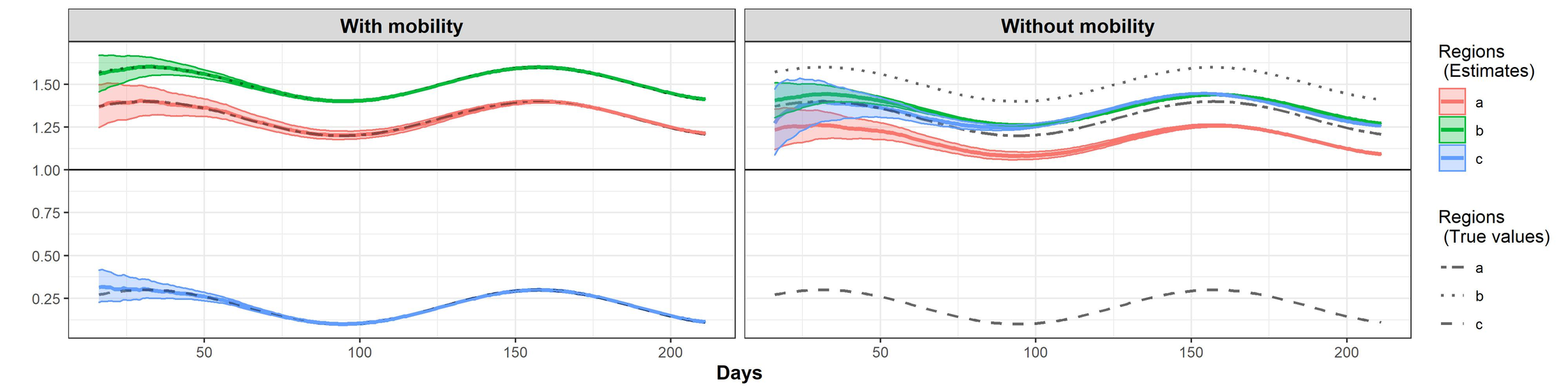

Supplement: S3 Fig — Solid lines are posterior means, along with the 95% credible bands (shaded). The results are summarized from Approach I with and without incorporating mobility information. (TIF) [file pcbi.1010434.s004.tif]

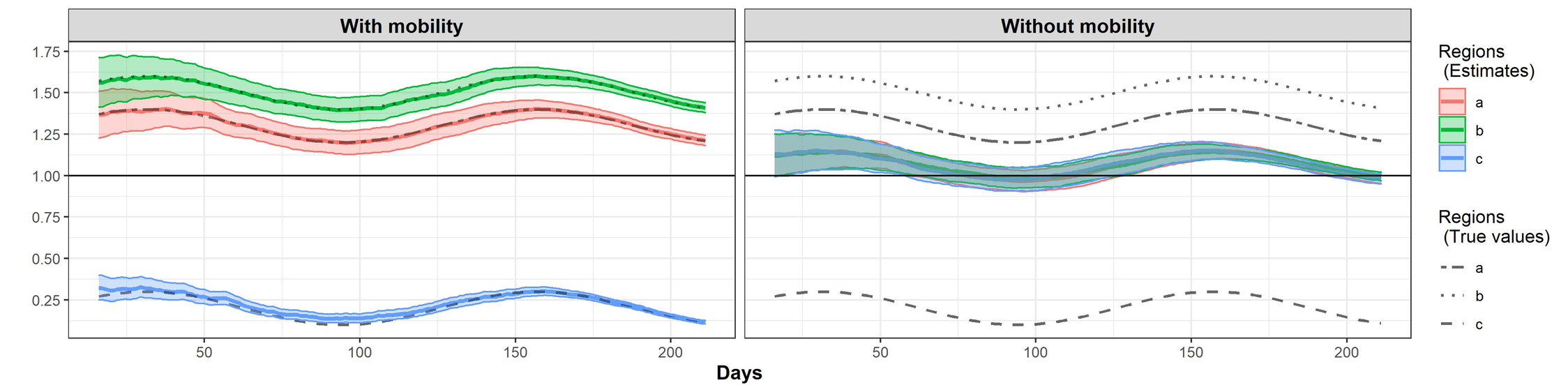

Supplement: S4 Fig — Solid lines are posterior means, along with the 95% credible bands (shaded). The results are summarized from Approach I with and without incorporating mobility information. (TIF) [file pcbi.1010434.s005.tif]

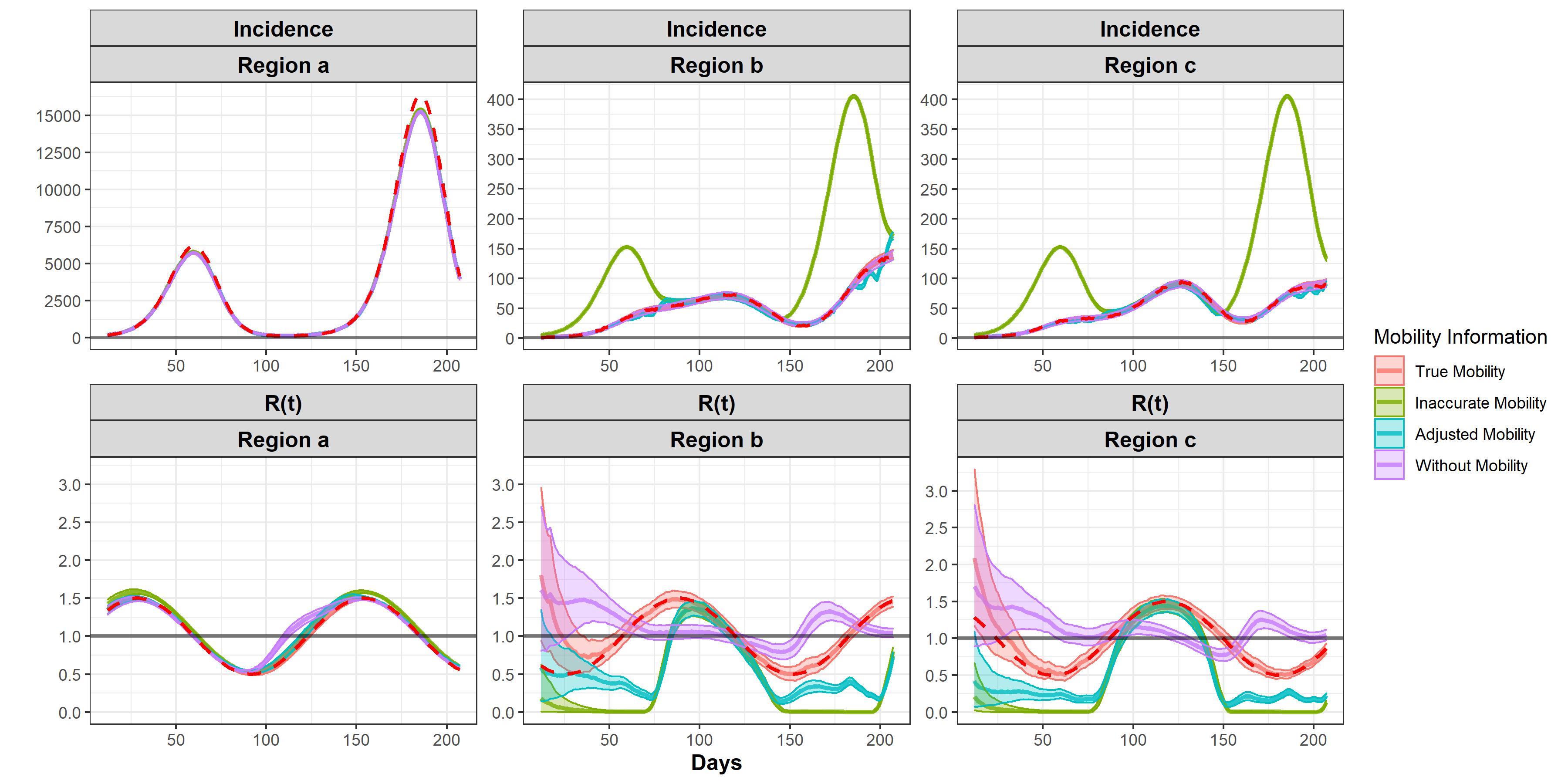

Supplement: S5 Fig — Solid lines are posterior means, along with the 95% credible bands (shaded), the color of solid lines represents the mobility information used in the model. Red dashed lines are means of N(t) and R(t) in the simulated data. (JPG) [file pcbi.1010434.s006.jpg]
